# Use of the prone position in pregnant women with COVID-19 or other health conditions

**DOI:** 10.1590/1518-8345.5181.3494

**Published:** 2021-11-08

**Authors:** Francisco Marcelo Leandro Cavalcante, Cristina da Silva Fernandes, Luanna dos Santos Rocha, Nelson Miguel Galindo-Neto, Joselany Áfio Caetano, Lívia Moreira Barros

**Affiliations:** 1 Universidade Estadual Vale do Acaraú, Centro de Ciências da Saúde, Sobral, CE, Brazil.; 2 Scholarship holder at the Fundação Cearense de Apoio ao Desenvolvimento Científico e Tecnológico (FUNCAP), Brazil.; 3 Universidade Federal do Ceará, Departamento de Enfermagem, Fortaleza, CE, Brazil.; 4 Scholarship holder at the Coordenação de Aperfeiçoamento de Pessoal de Nível Superior (CAPES), Brazil.; 5 Instituto Federal de Educação, Ciência e Tecnologia de Pernambuco, Campus Pesqueira, Pesqueira, PE, Brazil.; 6 Scholarship holder at the Conselho Nacional de Desenvolvimento Científico e Tecnológico (CNPq), Brazil.; 7 Universidade da Integração Internacional da Lusofonia Afro-Brasileira, Curso de Enfermagem, Redenção, CE, Brazil.

**Keywords:** Pregnant Women, Pregnancy, Coronavirus Infections, Adult Respiratory Distress Syndrome, Prone Position, Nursing, Mujeres Embarazadas, Embarazo, Infecciones por Coronavirus, Síndrome de Dificultad Respiratoria del Adulto, Posición Prona, Enfermería, Gestantes, Gestação, Infecções por Coronavírus, Síndrome do Desconforto Respiratório do Adulto, Decúbito Ventral, Enfermagem

## Abstract

**Objective:**

to analyze, in the scientific literature, the knowledge available on the use of the prone position in pregnant women diagnosed with COVID-19 or other health conditions.

**Method:**

an integrative literature review developed through the following guiding question: What is the scientific knowledge available on the use of the prone position in pregnant women with COVID-19 or other health conditions? The search for studies was carried out in eight databases.

**Results:**

using the prone position in pregnant women with Acute Respiratory Distress syndrome allowed for improvements in lung compliance and oxygenation. It also allowed reducing uterine compression on the maternal large vessels, and a reduction in blood pressure was observed in pregnant women with pre-eclampsia. The prone position was also safe in the surgical management of pregnant patients. In addition, the following conditions stood out as disadvantages related to the prone position in pregnant women: possibility of aortocaval compression, causing severe hypotension, and inability to easily monitor fetal status or to perform emergency Cesarean sections.

**Conclusion:**

the prone position was considered safe, reliable and comfortable for its use in the clinical management of pregnant women, where specific care measures must be taken to avoid compression of gravid abdomen, as well as fetal monitoring is important to detect placental circulation impairment.

## Abstract (es)

**Objetivo:**

analizar, en la literatura científica, el conocimiento disponible sobre el uso de la posición prona en gestantes diagnosticadas con COVID-19 u otras condiciones de salud.

**Método:**

revisión integradora de la literatura desarrollada a través de la siguiente pregunta orientadora: ¿Qué conocimiento científico está disponible sobre la aplicación de la posición prona en gestantes con COVID-19 u otras condiciones de salud? La búsqueda de estudios se realizó en ocho bases de datos.

**Resultados:**

colocar a las gestantes con Síndrome de Dificultad Respiratoria Aguda en posición prona permitió mejorar la distensibilidad pulmonar y la oxigenación. También permitió una reducción de la compresión uterina de los grandes vasos maternos y se observó una reducción de la presión arterial en gestantes con preeclampsia. La posición prona también demostró ser segura en el manejo quirúrgico de pacientes embarazadas. Además, se destacaron desventajas relacionadas con la posición prona en la gestante: posibilidad de que ocurra una compresión aortocava, que provoque hipotensión severa, y la imposibilidad de monitorear fácilmente el estado fetal o realizar una cesárea de emergencia.

**Conclusión:**

la aplicación de la posición prona para el manejo clínico de la gestante se consideró segura, confiable y cómoda, se deben implementar cuidados específicos para evitar la compresión del vientre materno y monitorear al feto para detectar si la circulación placentaria está comprometida.

## Abstract (pt)

**Objetivo:**

analisar, na literatura científica, o conhecimento disponível sobre a utilização da posição prona em gestantes diagnosticadas com COVID-19 ou outras condições de saúde.

**Método:**

revisão integrativa da literatura desenvolvida mediante a seguinte questão norteadora: Qual o conhecimento científico disponível sobre a aplicação da posição prona em gestantes com COVID-19 ou outras condições de saúde? A busca de estudos foi realizada em oito bases de dados.

**Resultados:**

a aplicação da posição prona em gestantes com Síndrome do Desconforto Respiratório Agudo permitiu a melhora na complacência pulmonar e na oxigenação. Também possibilitou redução da compressão uterina sobre grandes vasos maternos e foi observada redução da pressão arterial em gestantes com pré-eclâmpsia. A posição prona também se mostrou segura no manejo cirúrgico de pacientes grávidas. Ademais, destacaram-se como desvantagens relacionadas à posição prona em gestantes: a possibilidade de ocorrência de compressão aortocaval, causando hipotensão grave, e a incapacidade de monitorar facilmente o estado fetal ou realizar cesariana de emergência.

**Conclusão:**

a posição prona foi considerada segura, confiável e confortável para aplicação no manejo clínico de gestantes, na qual cuidados específicos devem ser tomados para evitar compressão do abdome gravídico, assim como é relevante a monitorização fetal para detectar comprometimento de circulação placentária.

## Introduction

SARS-CoV-2 had its first cases in Wuhan, China, where in late December 2019 a number of pneumonia cases caused by unknown etiologic agents were reported to the World Health Organization (WHO). In the following year, the problem became global, being declared as a pandemic in March 2020. COVID-19, a disease caused by SARS-CoV-2, can cause complications to the infected patient, such as Acute Respiratory Distress Syndrome (ARDS), sepsis, acute renal failure and cardiac dysfunction^(1)^.

Since notification of the first COVID-19 cases in the American Continent, more than 60,000 confirmed cases of the disease were identified among pregnant women, including 458 maternal deaths according to the Pan American Health Organization (PAHO), with more than half occurring in Brazil and Mexico^(2)^. Until December 16^th^, 2020, the Brazilian Ministry of Health recorded 4,564 hospitalization cases due to ARDS and 233 deaths among pregnant women with confirmed COVID-19 cases in the country, indicating a mortality rate of 86.2 deaths/100,000 inhabitants in this population^(3)^.

The high morbidity and mortality rate due to COVID-19 among Brazilian pregnant women can be related both to pathophysiological conditions inherent to the process of pregnancy and illness caused by infection with SARS-CoV-2, and to the chronic problems faced by Brazilian obstetric care - such as low quality and difficulty accessing emergency and high-complexity care^(4)^. Thus, it is understood that the assistance provided to pregnant women affected by the disease requires special care measures, in an attempt to preserve the best obstetric practices and to achieve positive maternal and fetal outcomes^(5)^.

Initially, pregnant women did not constitute a risk group for COVID-19, but they were later included in this classification. While the number of infections by the new coronavirus in this group is not higher when compared to the general population, current studies have pointed out an increased need for hospitalization in Intensive Care Units (ICUs) and for invasive ventilation and extracorporeal membrane oxygenation support, as well as a higher risk for premature birth and maternal death in the group of pregnant women, when compared to the non-pregnant population^(6-7)^.

In this context, it is important to note that the physiological changes that occur during pregnancy make pregnant women more vulnerable to serious infections. Elevation of the diaphragm, coupled with increased oxygen consumption, causes a reduction in pulmonary functional capacity during pregnancy, which increases complications in ARDS cases. In addition, pregnant women with ARDS have increased risk for Cesarean sections, premature births, decreased Apgar scores and low weight in their newborns. Even patients with COVID-19 who present mild symptoms have a high risk of developing ARDS and adverse pregnancy outcomes, with special attention to pregnant women with diabetes, cardiovascular diseases, obesity, pre-eclampsia or other complications/comorbidities^(4,8)^.

Improved respiratory patterns in patients with ARDS can be obtained with prone positioning, which increases ventilation homogeneity, since it decreases ventral alveolar distension and alveolar dorsal collapse to reduce the difference between the dorsal and ventral transpulmonary pressures, in addition to reducing compression of the lungs. The patient’s prone positioning in the bed must be implemented as early as possible, preferably within the first 24 or 48 hours, given the ARDS condition^(9)^.

Prone positioning, which consists in moving the patient from the supine position to lying face down, is a therapy used to increase survival probability in patients with COVID-19. The technique was first described as a treatment for Acute Respiratory Distress Syndrome (ARDS) in the scientific literature more than 40 years ago. The procedure was initially used as a last resource, when all other treatments have failed. However, recent findings suggest that the use of prone positioning must be included as part of the early treatment for severe ARDS^(10)^.

A number of studies show that the prone position, applied in an early and sustained way, can be a protective factor against mortality in patients with ARDS using invasive ventilation. However, its use in conscious patients does not yet present conclusive results, mainly in pregnant women^(11-12)^.

Thus, given the scientific gaps, the question on the use of the prone position in pregnant women with COVID-19 or other health conditions emerged. A randomized clinical trial conducted in Australia evidenced an improvement in systolic blood pressure in pregnant women with pre-eclampsia after prone positioning^(13)^. Seven pregnant women in urolithiasis crises in the first gestational semester underwent Percutaneous Nephrolithotomy with use of prone positioning and achieved good outcomes without complications^(14)^. The analysis of 22 clinical cases in a narrative review found that the prone position was feasible to perform lumbar disc hernia surgery in pregnant women during the third gestational trimester^(15)^.

Therefore, this study is justified by the need to foster prone positioning in patients with COVID-19 or other health conditions, especially for pregnant women, who represent a population with significant hints about the risks and complications arising from SARS-CoV-2. Thus, this study aimed at analyzing, in the scientific literature, the knowledge available on the use of the prone position in pregnant women diagnosed with COVID-19 or other health conditions.

## Method

### Study design

An integrative literature review developed through the following stages: elaboration of the research question; definition of eligibility criteria for the studies; search for publications in the scientific literature; data collection from the publications; critical analysis of the selected studies; discussion of the results; and presentation of the integrative review^(16)^.

The study guiding question was prepared using the Context, Population, Interest (PICo) strategy^(17)^, where the following was considered: P (Population): Pregnant women; I (Interest): Prone position; Co (Context): Pregnancy. Thus, the following question was defined: What is the scientific knowledge available on the use of the prone position in pregnant women with COVID-19 or other health conditions?

It was decided to include the “COVID-19” term in the guiding question so as to confer more emphasis to the content of this manuscript, which may facilitate retrieval of publications during the search for articles on the theme. On the other hand, the “other health conditions” term refers to any clinical or trauma complication leading the pregnant women to need prone positioning, being possible to infer that, when defining the term Pregnant women in the PICo strategy, it is implied that any health condition experienced during the gestational period would be obtained in the search.

### Period

Data collection was conducted from October to November 2020.

### Selection criteria

Selection of the studies took place according to the recommendations of the *Preferred Reporting Items for Systematic Reviews and Meta-Analyses* (PRISMA)^(18)^, by means of the following inclusion criterion: publications that addressed the use of the prone position in pregnant women, published without time and language restrictions. Review studies, theses, dissertations, editorials, annals and duplicate studies were excluded.

### Data collection

The following databases were used to search for publications: PubMed Central (PubMed/PMC), Scientific Electronic Library Online (SciELO), Scopus, *Literatura Latino-Americana e do Caribe em Ciências da Saúde* (LILACS), Web of Science, Cochrane, Cumulative Index of Nursing and Allied Health Literature (CINAHL) and Excerpta Medica dataBASE (EMBASE).

The search, screening, selection and analysis process of the studies was developed by two researchers independently and in a paired manner, in order to verify possible differences in the findings. It is worth noting that if there was disagreement between the two researchers in the selection of studies, a third reviewer analyzed the study and gave the final decision on its inclusion or not.

**Figure 1:**
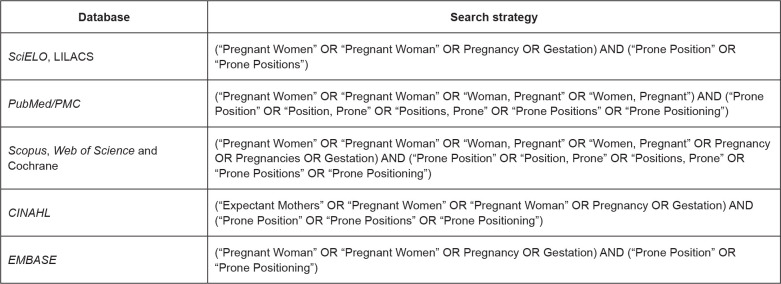
Search strategies and their respective databases. Sobral, CE, Brazil, 2020

To obtain the greatest number of possible results in each database, descriptors and keywords from the Medical Subjects Heading (MeSH), Descriptors in Health Sciences (*Descritores em Ciências da Saúde*, DeCS), EMBASE Heading Subjects (EMTREE) and CINAHL^®^ Headings were used, with which the search strategies described in Figure 1 were defined.

It was decided not to use terms related to COVID-19 in order to enable retrieval of a greater number of possible results and due to the need to expand the possibility of search for studies on the use of the prone position in women with other health problems. In addition to that, if the COVID-19 term was included in the search, it would be necessary to also include terms related to other clinical conditions, such as metabolic and cardiovascular diseases that may come to occur during this period. It is also worth noting that the Gestation and Pregnancy terms were also selected in order to increase the search results, which would not be possible with the restriction for terms such as risk pregnancy or pregnancy with comorbidities, for example.

### Data analysis

The studies found in the databases were exported to the *Mendeley* reference manager, whereby duplicate studies were excluded. Immediately after that, the titles and abstracts of the publications were read to select those that met the eligibility criteria, where the selected studies were read in full and those that did not answer the research question were excluded. Subsequently, the articles included in the final sample were fully analyzed through the use of a semi-structured instrument to obtain information considered relevant about the studies, such as: title; authors; year, language and country of publication; methodological aspects and main results evidenced, data that were grouped into descriptive charts.

In addition to that, the publications were classified into evidence levels according to the following convention: Level I - meta-analyses, controlled and randomized studies; Level II - experimental studies; Level III - quasi-experimental studies; Level IV - descriptive, non-experimental or qualitative studies; Level V - experience and case reports; and Level VI - experts’ opinion and consensus^(19)^.

### Ethical aspects

The ethical precepts set forth in Law No. 9,610/98 were followed, respecting the ideas, concepts and definitions of the authors of the studies selected for this review.

## Results

**Figure 2:**
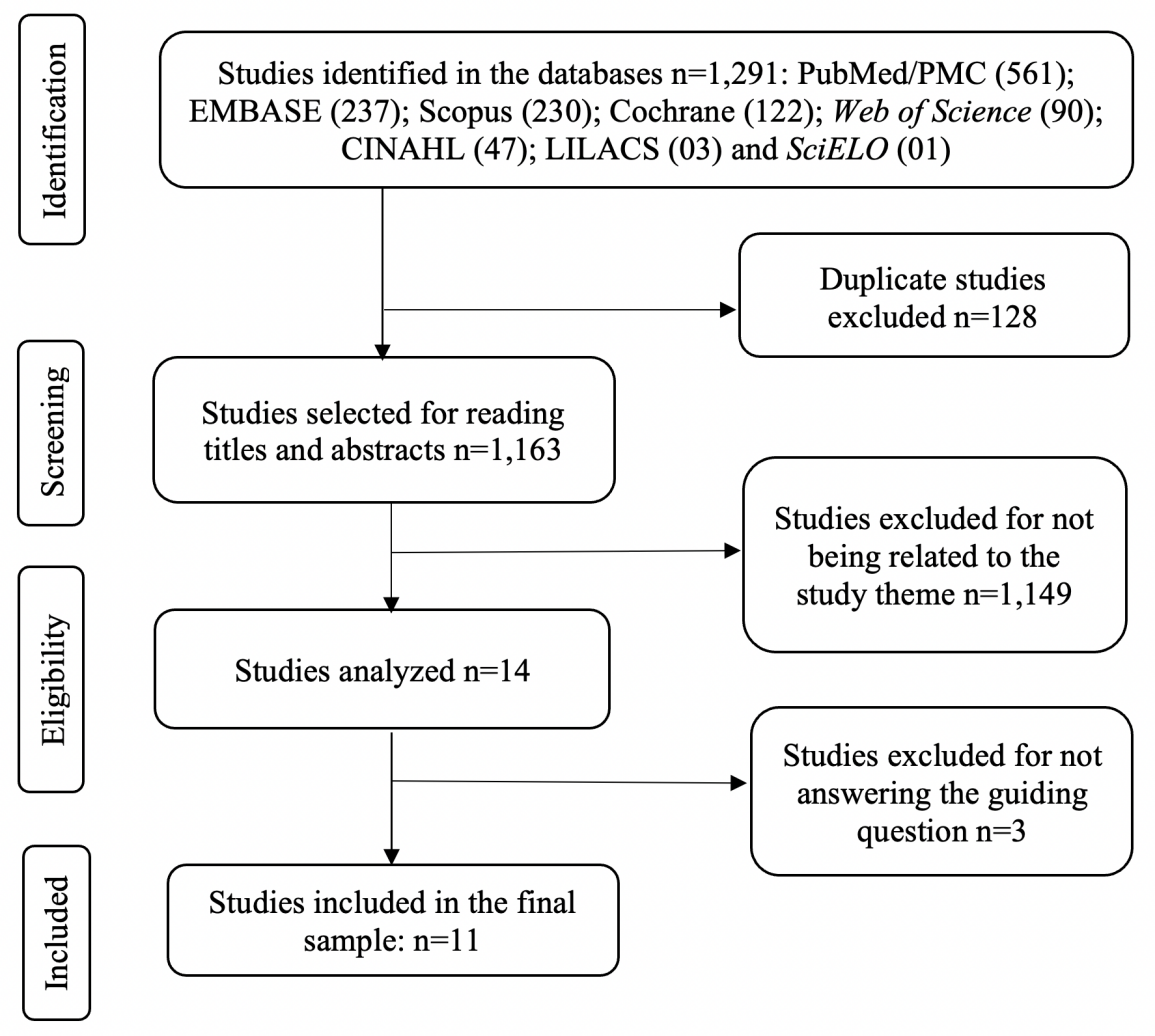
Search flowchart according to the PRISMA^(18)^ recommendations

A total of 1,291 publications were retrieved, of which 1,279 were excluded: 1,151 for not answering the guiding question and 128 for being duplicates. The final sample consisted of 11 studies. Figure 2 describes the process corresponding to the search and selection of studies.

The publications were predominantly from 2020 (36.3%), followed by 2014 (18.1%) and 2018, 2017, 2015, 2009 and 1998, with one (9.0%) publication each. All the studies were published in English. Regarding the country of origin, the United States, Italy and Australia predominated with two (18.1%) publications each, while Brazil, Japan, India, the United Kingdom and Germany contributed one (9.0%) publication each.

**Figure 3:**
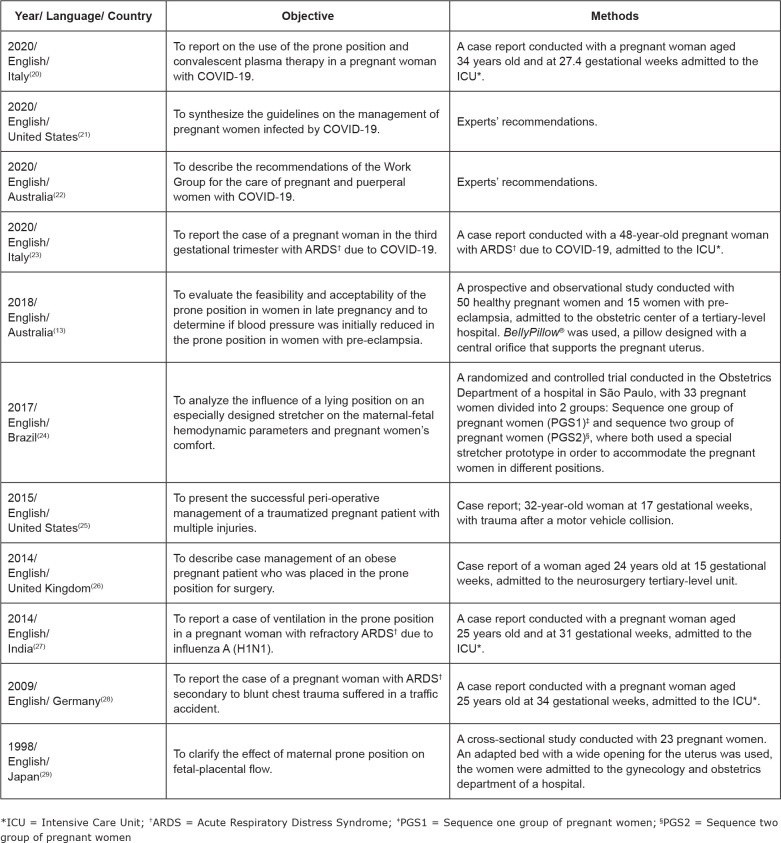
Description of the studies included in the review. Sobral, CE, Brazil, 2020

Studies of the case-report type prevailed (54.5%), followed by experts’ recommendations (18.1%). The cross-sectional study, randomized clinical trial and observational study types had one (9.1%) publication each. As for the level of evidence, Level V (n=6, 54.5%) was prevalent, followed by Level VI (n=2, 18.1%), Level IV (n=2, 18.1%) and Level II (n=1, 9.1%). Figure 3 shows the description of the studies.

**Figure 4:**
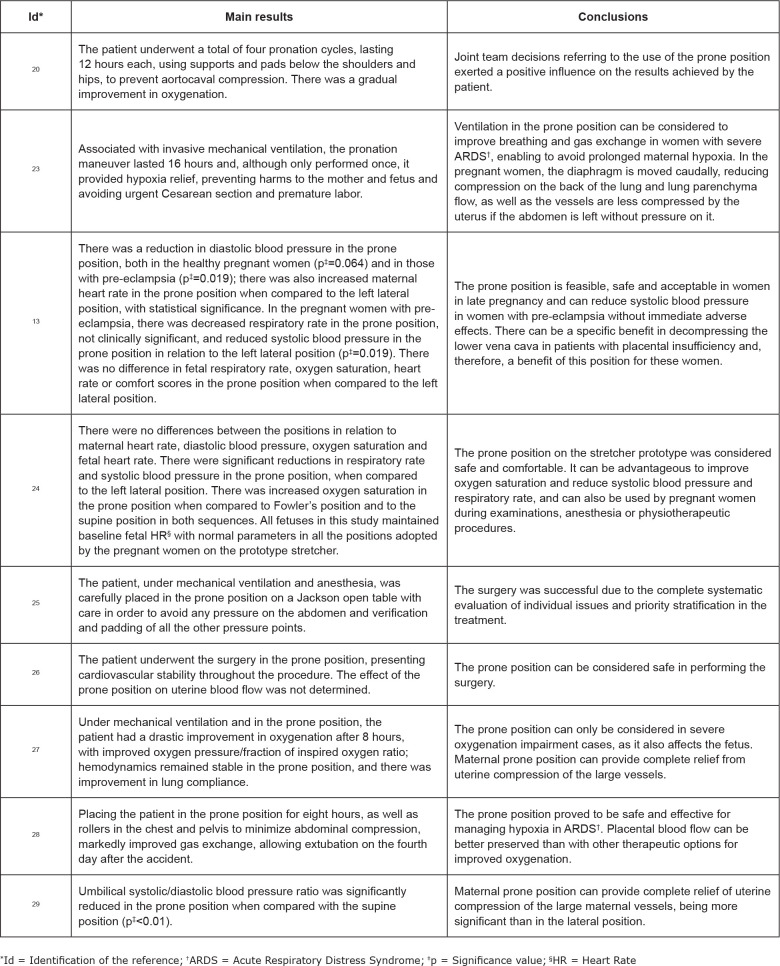
Description the main findings of the studies. Sobral, CE, Brazil, 2020

Figure 4 presents the main findings of the studies, with regard to the results and conclusions. Only two studies pointed the disadvantages related to using the prone position in pregnant women, in which they emphasized the possibility of aortocaval compression causing severe hypotension^(20)^; as well as the inability to easily monitor fetal status or to perform an emergency Cesarean section^(25)^. No study reported the use of protocols or checklists for the procedure.

**Figure 5:**
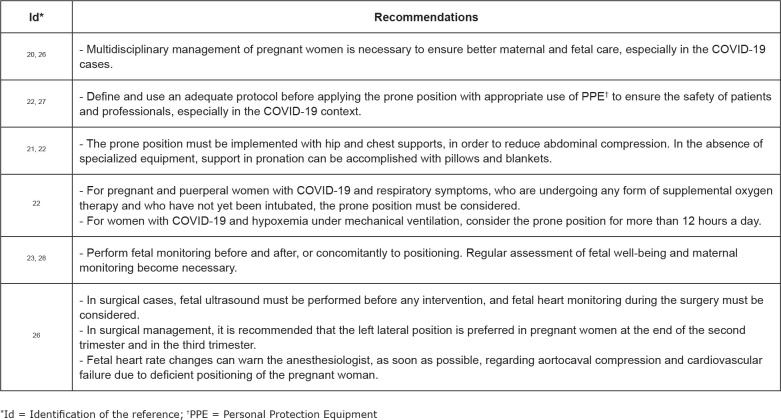
Recommendations for the use of the prone position in pregnant women. Sobral, CE, Brazil, 2020

By analyzing the studies, it was possible to list the recommendations for using the prone position in pregnant women, both in the COVID-19 cases and for other health conditions, as described in Figure 5.

## Discussion

This review enabled us to identify that most of the studies were published in 2020, which is justified due to the emerging need for production of knowledge on therapeutic methods for the treatment of patients who develop ARDS resulting from COVID-19, especially in cases of pregnant women. In this context, the prone position has shown to be safe, reliable and comfortable both for the clinical management of this vulnerable group, which presents severe ARDS cases related or not to the new coronavirus, and for surgeries.

The studies conducted in Australia and Brazil showed no significant changes in pregnant women in the prone position, referring to the diastolic blood pressure, respiratory rate, oxygen saturation and fetal heart rate parameters; in addition, a study reported increased maternal heart rate with statistical significance, although the etiology of this finding is not known for certain^(13-24)^. However, it is emphasized that, when applied correctly, the prone position will not result in adverse effects on the patient’s hemodynamics, but may induce improvements in the hemodynamic parameters due to its positive effects on the cardiac and pulmonary systems, which makes it fundamental to preserve venous return when this technique is employed^(30)^.

From this perspective, diverse evidence indicated that the prone position provided various beneficial effects when applied with different durations, both in conventional beds as in specially designed equipment, such as the *BellyPillow* ^®^, findings that signal this therapeutic method as a potential care strategy for pregnant women at different gestational weeks^(17-19,25,27)^.

In relation to the benefits in the respiratory and ventilation pattern when the prone position is applied during different periods of time, a number of studies emphasized the improvement of lung compliance with a progressive increase in oxygenation and oxygen saturation, decreased hypoxia, improved relative partial pressure of oxygen (PO_2_)/fraction of inspired oxygen (FiO_2_), improved gas exchange and flow displacement of the diaphragm, which enables a reduction in lung compression^(20-23,27-28)^. Therefore, this care tool can be an alternative in the treatment of the severe cases of pregnant women with COVID-19.

In this sense, for the management of pregnant women with COVID-19 who require supplemental oxygen support, it is recommended that the prone position be applied for more than 12 hours a day. In these cases, multidisciplinary management becomes necessary to ensure that effective teamwork is performed for the safe application of the prone position with the fewest possible adverse events, as well as it is necessary to continuously evaluate the clinical parameters through blood gas analysis^(20)^.

A descriptive study conducted by researchers from Houston, Texas, reported the development of a guideline and algorithm which included indications, contraindications, and step-by-step instructions for pronation of awake and intubated pregnant patients. The authors mentioned that admission of these clients with a severe respiratory condition requires multidisciplinary planning in order to prevent complications associated with the use of the pronation technique^(31)^. Thus, prone positioning may present positive results, as long as it is carried out by a trained team, by means of validated and adapted protocols to the structural and technological reality of the health institutions.

Furthermore, it was shown that the prone position in pregnant women can provide relief from uterine compression of the large maternal vessels, with preservation of placental blood flow, when compared to other positions. Such fact makes its application feasible, since it also does not compromise blood supply to the fetus, as there is no abdomen compression^(27,29)^.

In this sense, the occurrence of aortocaval compression stood out as a disadvantage of implementing the prone position. Thus, special care must be taken to avoid compression of the uterus over the maternal vessels, since this can cause a significant reduction in maternal cardiac output and systemic blood pressure, which, can consequently trigger severe hypotension, impaired uterine blood flow, and fetal distress^(20,26)^.

A reduction was observed in systolic blood pressure without immediate adverse effects on the pregnant women with pre-eclampsia, in which there may be a benefit resulting from decompression of the lower vena cava in patients with placental insufficiency^(13)^. Such findings can justify the implementation of the prone position as a complementary therapeutic tool in order to reduce blood pressure in pregnant women with pre-eclampsia, although more studies are needed to assess the best duration of this positioning and its short- and long-term effects on this group.

A number of studies carried out in the United States and in the United Kingdom also reported successful surgical management of pregnant patients in the prone position, in which, in one of the cases, there was maternal hemodynamic stability throughout the procedure^(26)^. Fetal cardiac parameters and uterine flow during the surgery were not evaluated; however, maternal stability throughout the procedure can suggest safety both for the pregnant woman and for the fetus. However, it is recommended that, although the prone position facilitates access to the surgical incision site, the lateral position can be considered to avoid uterine compression and possible complications for the fetus^(25-26)^.

It was also identified that, during the first and early second trimesters, the prone position for performing neurosurgery is safe, although the left lateral position is preferred with pregnant women in the late second trimester and third trimester. However, in the studies selected in this review, similar recommendations regarding the use of the prone position in the treatment of pregnant patients with COVID-19 were not highlighted, which is subjectable to new robust research studies aimed at evaluating the feasibility of this technique in pregnant women in the late second trimester and third trimester with respiratory failure related to infection by the new coronavirus.

In addition, although it is difficult to perform fetal assessment in the prone position, fetal ultrasound and fetal heart monitoring become necessary before and during any intervention, in order to early identify and intervene on any complication that may occur^(26)^.

Given the above, nurses, who are the professionals responsible for care planning and coordination, should propose comprehensive, safe and holistic assistance strategies to be developed in the peri-operative period, given the need to monitor the safety of the pregnant woman and to assess fetal vitality when the prone position is applied, given the possible risks of the technique in different care contexts.

Using the prone position for pregnant women represents a care strategy that requires special attention by the multiprofessional team, since its management can result in potentially avoidable complications. Consequently, the need to implement care measures to prevent adverse events related to this procedure stands out, such as worsening of gas exchange and of the ventilatory parameters, development of pressure injuries, and decannulation of the endotracheal tube in patients under invasive mechanical ventilation.

Another necessary strategy, not addressed in the studies that comprised the sample, is the application of a checklist during the care directed to the pregnant woman who needs pronation. Using checklists based on scientific evidence corroborates the prevention and reduction of care errors, as well as it facilitates communication among the professionals^(32)^. Such care tools must be properly implemented, concomitantly with permanent training of the health professionals, in order to ensure proper conduction of the procedure and patient safety during health care.

The following stand out as limitations of this review: the low level of evidence in most of the publications, as well as the absence of information, in most studies, related to the disadvantages or adverse events resulting from using the prone position in pregnant women, as well as the absence of studies that contemplated the elaboration, validation and evaluation of checklists to perform the procedure in this profile of patients.

As a contribution to the nursing and healthcare clinical practice, this study presents the availability of the benefits, risks, advantages and disadvantages of applying the prone position in pregnant women, especially in those with ARDS resulting from COVID-19 or other health conditions. It is worth mentioning that the research identified scientific gaps that may come to justify other studies and raise questions to be answered by means of other research methods.

## Conclusion

The prone position was considered safe, reliable and comfortable for its application in the clinical management of pregnant women, both in cases of ARDS related to COVID-19 infection, as well as when conducting surgical procedures. The following stood out as benefits of the prone position: improvement in the respiratory pattern, ventilation and gas exchange, as well as reduced uterine compression on the maternal vessels and reduced blood pressure in pregnant women with pre-eclampsia.

The specific care measures for the pregnant woman are related to gravid abdomen, which requires bone ends, thorax and pelvis to be padded, so that there is accommodation of the abdominal volume. In addition to that, fetal monitoring is relevant, which will allow inferences on placental circulation impairment.

Thus, it is believed that the prone position constitutes a potential therapeutic tool in the care of pregnant women, and it is therefore necessary that the health teams use protocols and checklists while implementing this procedure, in order to ensure safety both for professionals and for patients.

It is suggested to develop new randomized clinical trials to assess the best period of time for the application of the prone position in women at different gestational weeks, as well as its long term effects on blood pressure in pregnant women with pre-eclampsia. In addition to that, it is recommended to carry out methodological studies that seek to develop and validate protocols for the safe performance of this procedure in pregnant women, as well as systematic reviews aimed at classifying the level of evidence of the application of pronation in this population, in differentiated clinical contexts, especially in the COVID-19 pandemic scenario.
